# Mitochondrial membrane protein-associated neurodegeneration (mpan) in Denmark - prevalence, clinical features and molecular background

**DOI:** 10.1186/s13023-026-04661-3

**Published:** 2026-10-22

**Authors:** Simon Bauder Led Sørensen, Suzanne Granhøj Lindquist, Morten Dunø, Otto Mølby Henriksen, Annika Reynberg Langkilde, Søren Færgeman, Jørgen Erik Nielsen, Birgitte Rode Diness

**Affiliations:** 1 Department of Clinical Medicine, University of Copenhagen, Copenhagen, Denmark; 2 Department of Clinical Genetics, Copenhagen University Hospital Rigshospitalet, Copenhagen, Denmark; 3 Neurogenetics Clinic and Research Lab, Danish Dementia Research Centre, Department of Neurology, Copenhagen University Hospital Rigshospitalet, Copenhagen, Denmark; 4 Department of Clinical Physiology, Nuclear Medicine and PET, Copenhagen University Hospital Rigshospitalet, Copenhagen, Denmark; 5 Department of Radiology, Copenhagen University Hospital Rigshospitalet, Copenhagen, Denmark; 6 Department of Clinical Genetics, Aarhus University Hospital, Aarhus, Denmark; 7 Department of Molecular Medicine, Aarhus University Hospital, Aarhus, Denmark

**Keywords:** Mitochondrial membrane protein-associated neurodegeneration, MPAN, C19orf12, Neurodegeneration with brain iron accumulation, NBIA, Spastic paraplegia type 43, SPG-43

## Abstract

**Background:**

Mitochondrial membrane protein-associated neurodegeneration (MPAN) is an ultra-rare neurodegenerative disease belonging to a group of diseases known as neurodegeneration with brain iron accumulation (NBIAs). Clinical findings include gait disturbances, spasticity, and cognitive impairment, as well as iron deposition in the basal ganglia. The disease is typically inherited in an autosomal recessive pattern and caused by pathogenic variants in the *C19orf12* gene, which encodes a mitochondrial membrane protein, the function of which is not fully understood.

**Objective:**

This nationwide study aimed to identify and describe MPAN patients in Denmark, focusing on the prevalence, clinical features, and pathogenic variants. Additionally, the study sought to further elucidate the genotype-phenotype spectrum of MPAN with additional cases that expand understanding of disease characteristics associated with *C19orf12* variants.

**Methods:**

A case-based approach was applied in the description of the clinical and genetic features of the included patients. Three patients were identified at Copenhagen University Hospital, Rigshospitalet, and one patient at Aarhus University Hospital, through a Danish national network, NeuroGENet. Clinical data was extracted from health records with patient consent.

**Results:**

The prevalence of MPAN in Denmark was estimated to be 1/1,500,000. Four patients with biallelic pathogenic variants in *C19orf12* were identified, including two siblings. All patients’ parents were consanguineous of Middle Eastern backgrounds. Of the two patients, homozygous for c·187 G > C, p.(Ala63Pro), one showed no signal changes on MRI of the brain, while the other did reveal discreet hypointensities, most likely representing iron. Both patients had milder phenotypes. Two patients were homozygous for c·32C > T, (p.Thr11Met) and had more severe symptoms with cognitive impairment and iron deposition on MRI.

**Discussion and conclusions:**

The prevalence of *C19orf12*-related diseases is considered low among the ethnic Danish population, in comparison to other published estimates.

The identified patients homozygous for c·187 G > C, p.(Ala63Pro) tend to have milder phenotypes and show a lesser tendency to brain iron accumulation. Given these findings and the broad clinical spectrum of patients with pathogenic *C19orf12* variants, it may be more appropriate to classify patients as being on a *C19orf12*-disease-spectrum rather than belonging to the group of NBIAs.

**Supplementary Information:**

The online version contains supplementary material available at https://doi.org/10.1186/s13023-026-04661-3.

## Background

Mitochondrial membrane-protein associated neurodegeneration (MPAN) is an ultra-rare neurogenetic disorder clinically characterized by progressive neurological symptoms often including gait difficulties, spasticity, dystonia, optic atrophy, and cognitive decline. The disease belongs to a group of disorders known as neurodegeneration with brain iron accumulation (NBIA) and MPAN is also referred to as NBIA type 4 [1].

MPAN was first defined in 2011 in a cohort of Polish NBIA patients who had pathogenic variants in the *C19orf12* gene [2]. The gene encodes a small mitochondrial membrane protein, the function of which is still unknown, although it is thought to be involved in lipid homeostasis, mitochondrial function, and autophagy [3, 4].

The prevalence of NBIA disorders has been estimated to be 1–3/1,000,000 globally, with approximately 15,000–20,000 affected individuals worldwide [5], while MPAN is estimated to account for 5–30% of all NBIA cases [6–8]. In Denmark, the prevalence of MPAN is currently unknown.

Initial symptoms can vary, but gait difficulties with onset in childhood to early adulthood are common initial findings, although later age at onset has been described [9]. The diagnosis is made based on clinical findings, genetic testing, and iron accumulation in the basal ganglia, indicated on MRI. Progression is generally slow compared to other NBIA disorders, and patients live well into adulthood. The terminal stages include severe cognitive decline, dysphagia, and bladder- and bowel-incontinence. The cause of death is often respiratory complications such as aspiration pneumonias [1].

The inheritance pattern is predominantly autosomal recessive (AR), but autosomal dominant (AD) inheritance has been reported. With rare exceptions the pattern of inheritance does not predict the clinical expression [10]. The most frequent pathogenic *C19orf12* variants associated with AR inheritance, are the Polish founder variant, c.204_214del, p.(Gly69Argfs*10), and the Turkish founder variant, c.32C > T, p.(Thr11Met) (NM_001031726.3) [11]. While most MPAN patients have onset of symptoms in childhood, adult onset of symptoms has been associated with the c.32C > T, p.(Thr11Met) variant [12]. Additional clinical differences include parkinsonism, which is a more prevalent initial finding among patients homozygous for c.32C > T, p.(Thr11Met). Rapid disease progression is reported to be more common among patients homozygous for c.32C > T, p.(Thr11Met) compared to patients homozygous for c.204_214del, p.(Gly69Argfs*10) [11–13].

Spastic paraplegia type 43 (SPG-43), a rare type of hereditary spastic paraplegias (HSP) has been associated with homozygosity for c.187 G > C, (p.Ala63Pro), which is predicted to affect the transmembrane domain of *C19orf12*. This variant was initially suspected of causing a milder phenotype, involving gait difficulties and spasticity, but no neurodegeneration nor brain iron accumulation [14]. However, as more cases of c.187 G > C, (p.Ala63Pro) patients were identified who showed brain iron accumulation and clinical findings indicative of a typical MPAN phenotype, the hypothesis of this variant being associated with isolated spastic paraplegia was disproved [15–17].

Treatment of MPAN is currently symptomatic [1].

## Methods

### Aim

The aim of this study was to identify Danish patients with *C19orf12*-defects and thereby estimate a prevalence of MPAN in Denmark. We wanted to provide a thorough clinical and genetic description of the identified patients, thereby contributing with additional cases and new insights into the discussion of MPAN and the *C19orf12* gene. Additionally, we wanted to examine the existing literature to gain an overview of the current *C19orf12* variants and phenotypes to establish whether our findings might be able to tip the scales in the genotype-phenotype discussion.

### Setting

The healthcare system in Denmark is publicly financed and freely available for all inhabitants, which results in most diagnostics, treatments and procedures being facilitated through the public healthcare system [18].

As of 2022, the Danish National Genome Center (NGC) has facilitated whole-genome-sequencing (WGS) to patients fulfilling a set of predefined criteria of of neurological symptoms where a genetic cause is suspected [19].

### Identification of Danish patients with pathogenic C19orf12 variants

A total of four patients with pathogenic *C19orf12* variants were identified. Three patients were identified at Rigshospitalet, Copenhagen University Hospital, two of which were diagnosed using WES and one using WGS facilitated by the NGC. Another patient, diagnosed at Aarhus University Hospital, was identified through the Danish collaborative national network called Genetic Expert Networks (GENets), which facilitates collaboration between all clinical genetics departments in the country [20].

All patients signed a written informed consent, allowing inclusion in this project.

Variants are described according to NM_001031726.3

## Results

### Patient I

Twenty-one-year-old female of Lebanese heritage, born to parents who are distantly related.

The patient presented 12 years old with gait disturbances. The waddling gait had been slowly developing for a few years.

The gait disturbances progressed slowly with Trendelenburg gait, positive Gowers sign, bilateral atrophy of the lower limbs, pes cavus and difficulties walking on toes and heels. She also began developing proximal muscle weakness in her shoulders and weakness in hands, although the weakness was most significant in her legs. Tendon reflexes were found to be normal, but plantar responses were atypical.

**Fig. 1:**
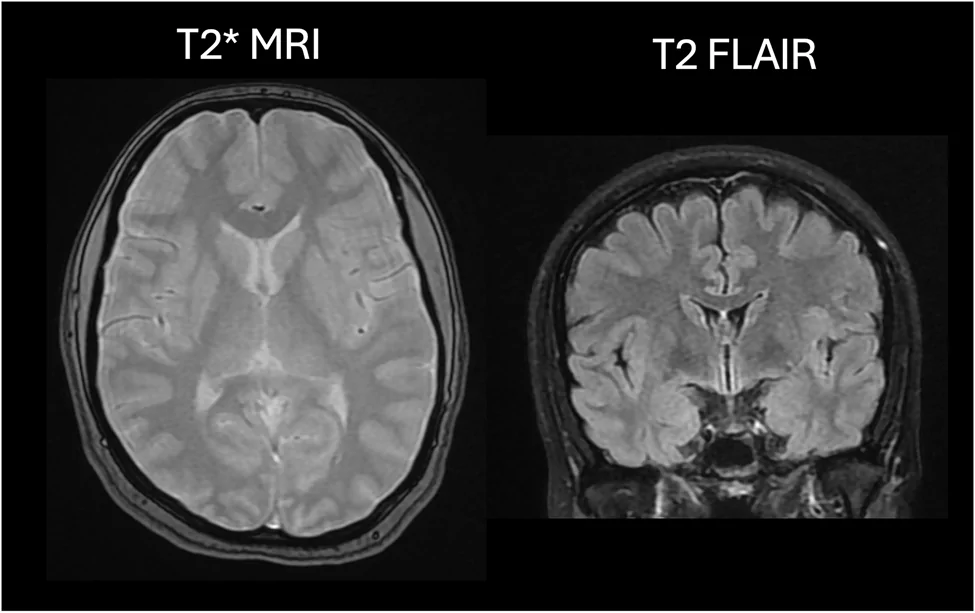
Patient I. Left axial T2* MRI and right coronal T2 FLAIR MRI. Note normal signal in basal ganglia not suggestive of iron accumulation, see Figs. 3 and 5. for comparison

Five years after onset, the patient was found to be homozygous for the known pathogenic c.187 G > C, p.(Ala63Pro) variant in *C19orf12*. She showed no psychiatric manifestations, brain iron accumulation on MRI (see Fig. 1) or bladder- and bowel symptoms, and the patient had normal vision, even though bitemporal papillary atrophy was found.

**Fig. 2:**
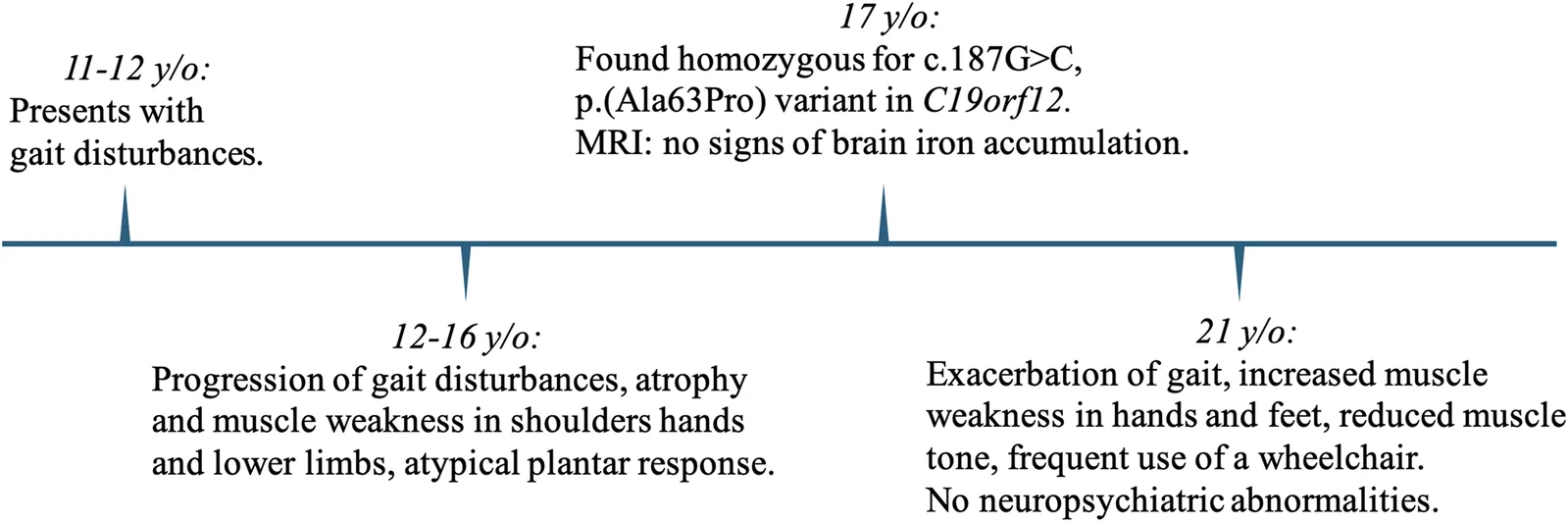
Timeline, patient I. Overview of the disease progression of patient I

By the most recent clinical control at age 21, the patient’s gait and muscle weakness had worsened, and she was using a wheelchair most of the time. She had muscle atrophy affecting hands and feet as well as claw hands bilaterally and generally reduced muscle tone (see video, additional file 1). However, she still showed no signs of neuropsychiatric abnormalities and was undergoing education. A timeline of the disease progression can be seen in Fig. 2.

### Patient II

Twenty-year-old male and the brother of patient 1.

He was diagnosed with ADHD at the age of five and treated with methylphenidate. He attended a special school and was found to have an IQ of 70 at age 15.

At 19 years, he was seen by a neurologist because of gait disturbances, reduced speed of gait and muscle strength, particularly in his right arm, progressing over the previous couple of years. His sister (patient 1) had recently been diagnosed with MPAN and upon genetic investigation, he was also found to be found homozygous for the pathogenic variant c.187 G > C, p.(Ala63Pro) in *C19orf12*.

The patient had severe generalized muscle weakness, particularly in shoulders, fingers, and lower limbs. At examination he showed patellar hyperreflexia, crossed adductor reflexes and weak Achilles reflexes. He had a broad, ataxic gait, and was able to walk on toes but not heels, nor on a line (see video, additional file 2).

A neuropsychological evaluation revealed a below average intelligence with a slow mental tempo, but with normal recognition of emotion, neutral mood, and normal insight of his own disease. All findings were assumed to be compatible with a low baseline intelligence level.

**Fig. 3:**
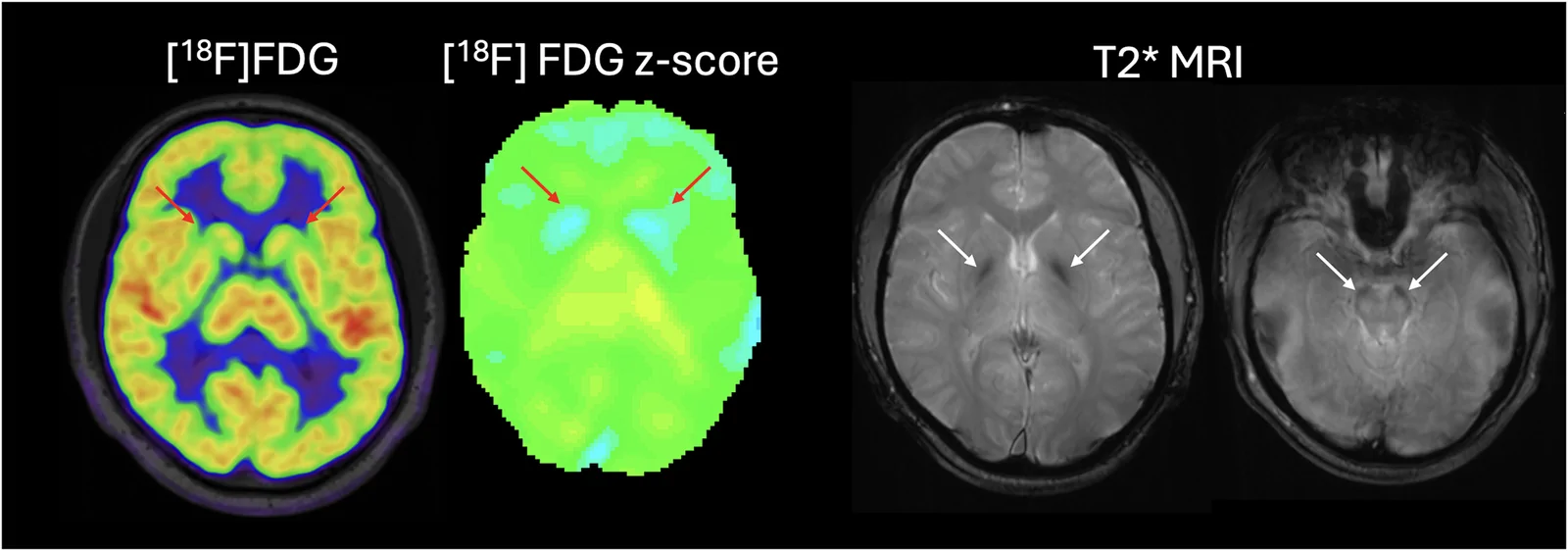
Patient II. Axial [^18^F]FDG PET and corresponding z-score at the level and basal ganglia shows mild hypometabolism in head of caudate nucleus bilaterally (red arrows). T2* MRI shows iron accumulation in globus pallidus and substantia nigra (white arrows)

**Fig. 4:**
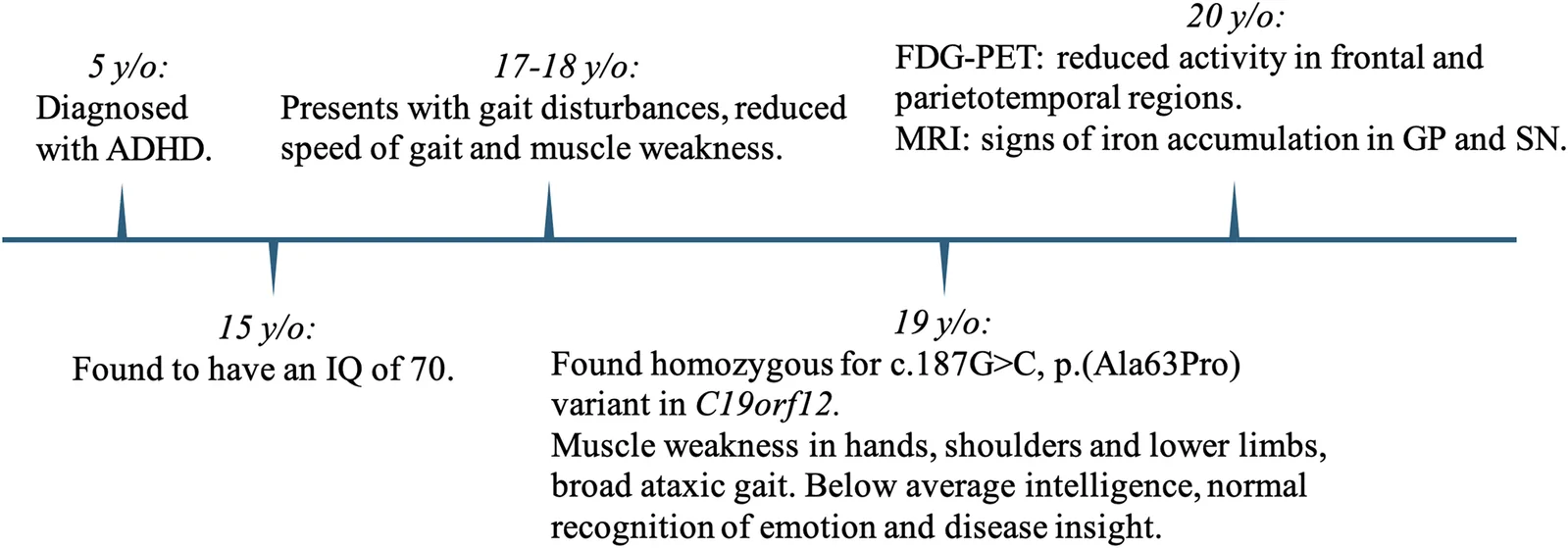
Timeline, patient II. Overview of the disease progression of patient II

A hybrid [18F]-fluorodeoxyglucose (FDG) positron emission tomography/magnetic resonance imaging (PET/MRI) of the brain (see Fig. 3) revealed slightly reduced metabolic activity in frontal and parietotemporal cortical regions, most pronounced left sided. Reduced activity was also seen in the heads of the caudate nuclei with slight progression for two years. The MRI revealed discreet hypointensities in the globus pallidus (GP) and substantia nigra (SN) likely due to iron accumulation. A timeline of the disease progression can be seen in Fig. 4.

### Patient III

Twenty-nine-year-old male, born to consanguineous parents of Turkish descent.

Initial symptoms began at 20–21 years of age and progressed gradually until he was neurologically assessed at age 26. Clinical examination showed atrophy and muscle weakness of his hands and impaired fine motor skills. He had gait difficulties with a drop-foot-like gait, pes cavus and left sided hyperreflexia.

The patient’s symptoms progressed over the following two years, experiencing more fatigue, atrophy, and weakness. He appeared cognitively slower and began to struggle finding words. A neuropsychological evaluation revealed memory-loss and reduced cognitive tempo.

**Fig. 5:**
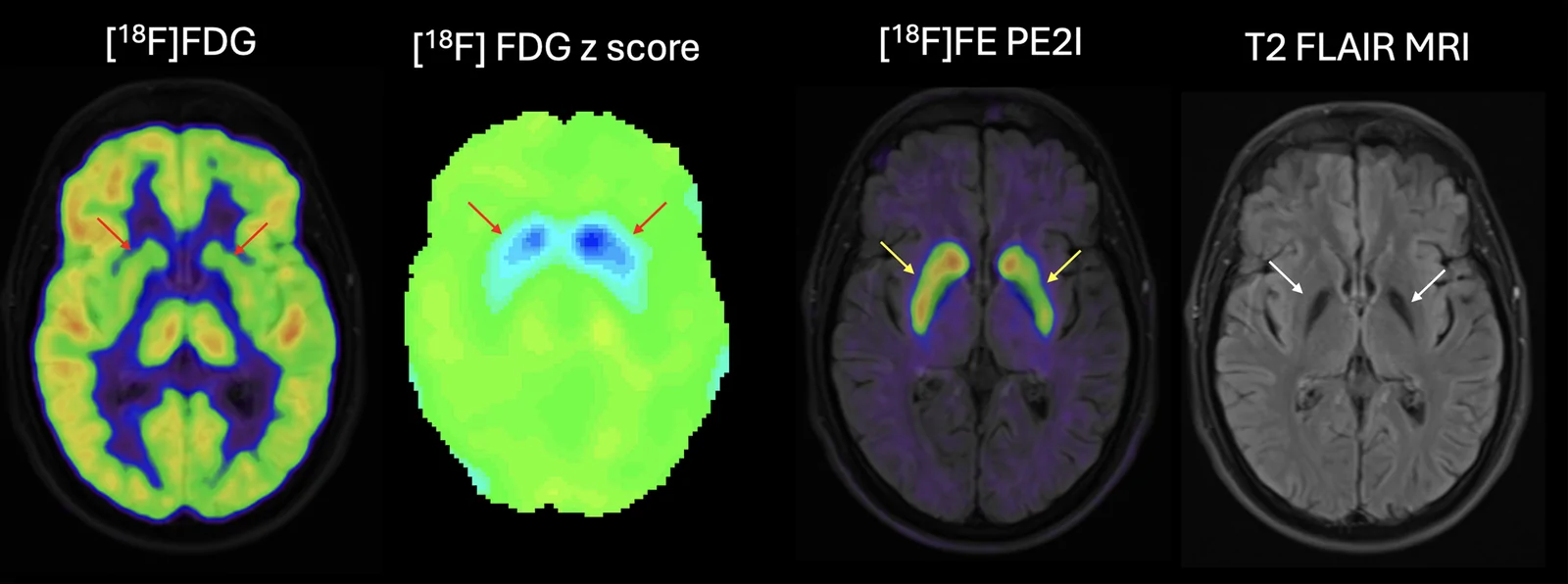
Patient III [^18^F]FDG PET and corresponding z-score shows moderate hypometabolism in anterior striatum bilaterally (red arrows) [18]. F]FE PE2I PET revealed decreased binding in both putamen and caudate nucleus, particularly in anterior putamen and more pronounced on the left side. T2 FLAIR MRI shows signs of iron accumulation globus pallidus (white arrows)

Two years after initial onset of symptoms, genetic analysis revealed the patient to be homozygous for c.32C > T, p.(Thr11Met), and to have pronounced presumed iron accumulation in the putamen and hypometabolism on PET-MRI in the anterior striatum bilaterally (see Fig. 5). No signs of optic atrophy or impaired vision were found.

**Fig. 6:**
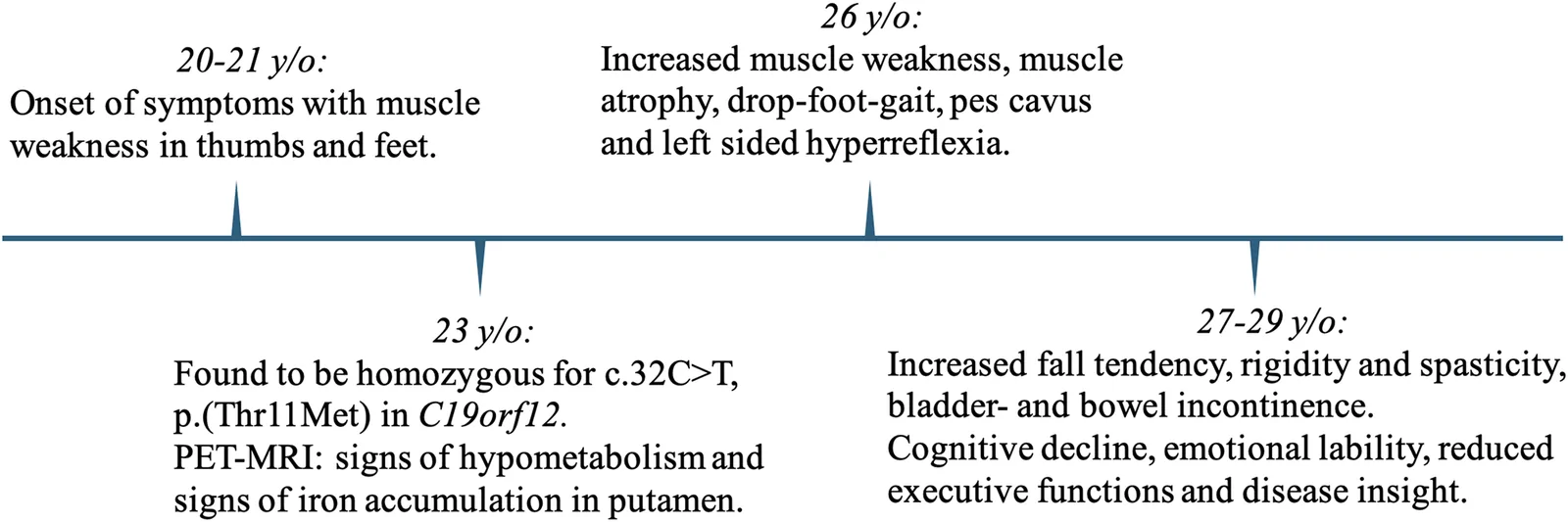
Timeline, patient III. Overview of the disease progression of patient III

The patient progressed rapidly, experiencing frequent falls, and showing rigidity, spasticity as well as reduced executive functions, attention, and disease insight. By the latest clinical control, he appeared emotionally labile and had begun experiencing bladder- and bowel incontinence. A timeline of the disease progression can be seen in Fig. 6.

### Patient IV

Forty-two-year-old female born in Syria from consanguineous parents.

At 25 years old she developed impaired fine motor skills in the hands. She had surgery under the suspicion of carpal tunnel syndrome and was subsequently referred to a neurological evaluation due to progressive loss of fine motor skills and reduced strength in her hands.

The patient was subjected to torture in Syria at age 31 and subsequently fled to Denmark. She was then admitted to a psychiatric ward, where she was evaluated for depression, memory loss and PTSD.

Neurophysiological testing showed neurogenic changes and was suggestive of a multifocal neuropathy. An MRI of the brain conducted at the age of 39 revealed presumed iron accumulation in the basal ganglia. Based on this finding her neurologist suspected an NBIA and referred her to molecular genetic analysis.

Whole genome sequencing identified homozygosity for the c.32C > T, p.(Thr11Met) variant.

**Fig. 7:**
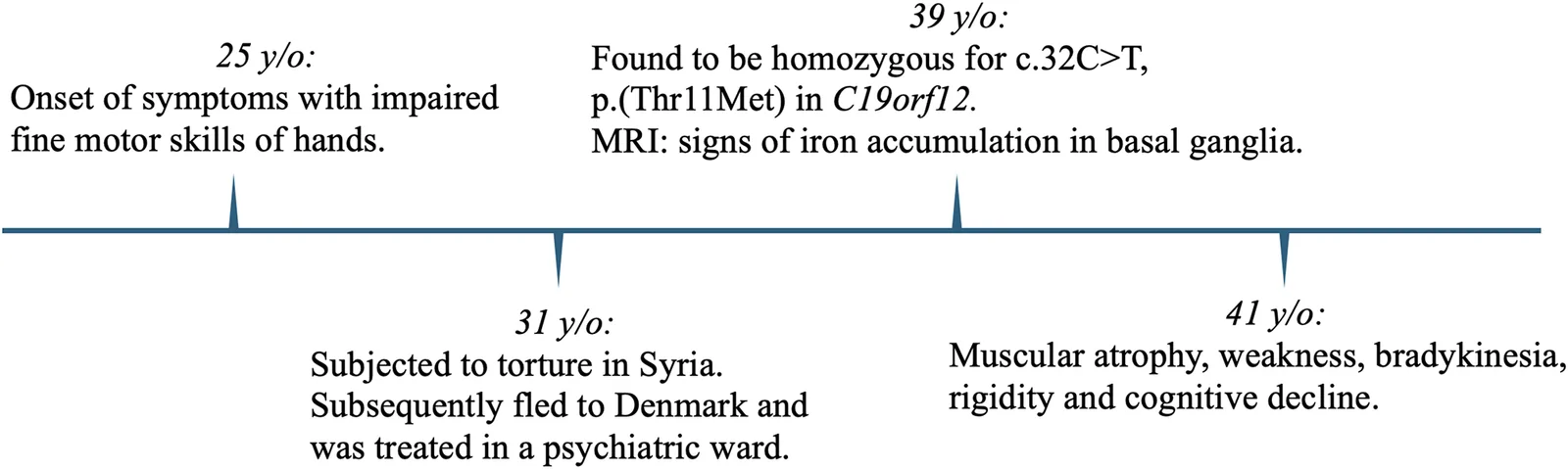
Timeline, patient IV. Overview of the disease progression of patient IV

At her latest clinical examination, at age 41, she had muscular atrophy, weakness, bradykinesia and rigidity, as well as cognitive decline. A timeline of the disease progression can be seen in Fig. 7.

### Overview of the described patients

### Clinical videos of patients I and II

Videos of the clinical findings seen in patients I and II can be found in additional files 1 and 2, respectively.

### The prevalence of C19orf12-related diseases in Denmark

The population in Denmark is 5,998,000 as of March 2025 [21]. Thus, the prevalence of MPAN and other *C19orf12*-related diseases in Denmark can be calculated to be **1/1,500,000**.

## Discussion

### The prevalence of C19orf12-related diseases in Denmark

The prevalence of *C19orf12*-related diseases in Denmark is estimated to be 1/1,500,000.

All known patients diagnosed with MPAN/*C19orf12*-related diseases in Denmark were identified and described. The national network, neuroGENet was used to ensure that all Danish patients with variants in *C19orf12* were identified. We therefore believe the identification of Danish patients, who have received a diagnosis due to pathogenic variants in *C19orf12* to be complete.

Furthermore, Denmark’s universal healthcare system ensures widespread access to diagnostic procedures across income levels, making it unlikely that a severe neurological condition like MPAN would remain undiagnosed in advanced stages of disease, despite some geographical healthcare disparities. If Danish MPAN patients with milder symptoms were to exist, it is reasonable to assume that these cases would have been found through the nationwide WGS-based diagnostic approach, which is applied for patients with considerably milder symptoms than those seen in MPAN, however, the possibility cannot be definitively excluded.All the identified cases have a Middle Eastern background and consanguineous parents.

MPAN is thought to make up 5–30% of NBIA disorders, which have an estimated prevalence of 1–3/1,000,000 [6, 7, 8]. Using this estimate, MPAN would be expected to have a prevalence ranging from 1/1,000,000 to 1/20,000,000.

Using gnomAD’s genetic prevalence estimator (GeniE) an MPAN prevalence of 1/15,782,085 among the European (non-Finnish) population, can be calculated [22, 23].

Our estimated prevalence of 1/1,500,000 lies within the uncertain range described in the literature between 1/1,000,000 and 1/20,000,000 and is significantly higher than the GeniE estimation, which is likely due to all identified patients being of Middle Eastern background.

### Genotype-phenotype correlations

In this study, two of the described patients are homozygous for c.187 G > C, (p.Ala63Pro), while the other two cases are homozygous for c.32C > T, (p.Thr11Met). This provides an opportunity to compare the associated phenotypes of the two homozygous variants.

Our clinical findings, as summarized in table 1, seem to align with previously reported cases. Patients 1 and 2, showing an HSP/SPG43-like-phenotype, have gait difficulties, spasticity, muscle atrophy and weakness with no signs of cognitive decline, or psychiatric symptoms. Of note, patient 1 shows no signs of iron accumulation, while patient 2 seem to have developed iron accumulation after onset of symptoms. The age at onset is similar between the two patients from early to late teens. It is not possible to know whether the presence of low IQ and ADHD in patient 2 is due to the *C19orf12* variant or other factors. The fact that the disorders were present prior to onset of symptoms might suggest that the two diagnoses are unrelated.

**Table 1:** Overview of all clinical and paraclinical findings in the four identified MPAN/SPG43 patients in Denmark

Patient number	I	II	III	IV
Family history	Consanguineous parents of Lebanese heritage Proband’s brother (patient II) diagnosed with SPG-43.	Consanguineous parents of Lebanese heritage. Proband’s sister (patient I) diagnosed with SPG-43.	Consanguineous parents. The patient is the proband of the family.	Consanguineous parents of Syrian heritage. The patient is the proband of the family.
Patient’s medical history	None prior to onset of symptoms	ADHD, low IQ	None prior to onset of symptoms.	Head trauma followed by cognitive impairment. Subsequent PTSD and depression.
Age at last examination	21 years	20 years	29 years	41 years
Age at onset	11–12 years	17–18 years	20–21 years	25 years
Symptoms at onset	Waddling gait.	Gait disturbances, reduced speed of gait and strength.	Atrophy of thenar, reduced thumb strength, reduced dorsal flexion in the ankles.	Reduced fine motor skills followed by tremor of both hands. Atrophy of thenar muscles bilaterally.
Disease duration	9 years	2 years	8 years	14 years
Pathogenic variant	Homozygous *C19orf12*-variant: c.187 G > C, p.(Ala63Pro)	Homozygous *C19orf12*-variant: c.187 G > C, p.(Ala63Pro)	Homozygous *C19orf12*-variant: c.32C > T, p.(Thr11Met)	Homozygous *C19orf12*-variant: c.32 C > T, p.(Thr11Met)
Phenotype	Spastic paraplegia type 43	Spastic paraplegia type 43	MPAN / NBIA 4	MPAN / NBIA 4
Gait abnormalities	++	+	+	+
Akinesia / hypokinesia	–	–	+	+
Rigidity	–	–	++	+
- Upper extremities	–	–	++	+
- Lower extremities	–	–	+	–
Spasticity	+	–	++	–
- Upper extremities	–	–	+	–
- Lower extremities	+	–	++	–
Tendon reflex abnormalities	++	++	++	–
- Upper extremities	Hyporeflexia	Hyperreflexia	Hyperreflexia	Normal
- Lower extremities	Patellar hyperreflexia, crossed adductor reflexes, no Achilles reflex, extensive plantar response	Patellar hyperreflexia, crossed adductor reflexes, weak Achilles reflex, plantar withdrawal response	Hyperreflexia, extensive plantar response	Normal
Muscle weakness	++	++	++	
- Upper extremities	+	++	++	+
- Lower extremities	++	+	++	–
Muscle atrophy	++	++	++	
- Upper extremities	++	++	++	++
- Lower extremities	++	+	+	–
Ataxia	–	+	–	–
Dysphagia	–	–	+	–
Dysarthria	–	–	+	–
Hypomimia	–	–	+	–
Bladder- and bowel incontinence	–	–	+	–
Cognitive decline	–	–	+++	++
Psychiatric symptoms	–	–	+	+
Apathy	–	–	+	–
Vision abnormalities	Impaired color vision	–	Disrupted eye movements	–
Brain MRI findings	Normal with no signs of iron accumulation	Symmetric presumed iron accumulation in the GP and SN PET-MR: Unspecific reduced metabolic activity.	Iron accumulation in the putamen PET-MR: Reduced metabolic activity in anterior striatum bilaterally	Symmetric iron accumulation in the basal ganglia, particularly in the GP
EMG / ENG	Chronic neuropathic changes	ND	Neurogenic changes	EMG/ENG: neurogenic changes suggestive of MND
Muscle biopsy	Unspecific signs of myopathy and vacuoles	ND	ND	ND
Eye fundus photography	Bitemporal papillary atrophy	ND	Normal	ND
Additional tests	None	Neuropsychological evaluation: below average intelligence, reduced mental tempo, normal emotion recognition and insight in own disease.	Neuropsychological evaluation: cognitive deficits in attention, memory, tempo, learning and executive functions. Normal CSF examination. Whole body PET-CT with no pathological findings.	Neuropsychological evaluation: Progressive decline in working memory and overall cognitive function, particularly within attention and executive functions. Visuospatial processing is severely impaired. PET-CT: Asymmetrical loss of PE2I binding in the anterior putamen suggestive of neurodegenerative disease.

GP = Globus pallidus SN = Substantia nigra ND = Not done

EMG/ENG = Electromyography/electroneurography MND = Motor neuron disease

PE2I = ^18^F-FE-PE2I (a dopamine transporter ligand used in PET imaging)

Patients 3 and 4 have the classical MPAN phenotype associated with c.32C > T, (p.Thr11Met), including a similar age at onset, ranging from early- to mid-twenties, impairment of fine motor skills as an initial symptom and progression of cognitive decline. Additionally, patients 3 and 4 had brain iron accumulation, and show a more rapid disease progression compared to patients 1 and 2.

Despite the age differences of the four identified patients, a phenotypical difference between the two identified pathogenic variants in *C19orf12* is apparent. Even though the existence of a milder and atypical presentation of c.187 G > C, p.(Ala63Pro) has been disproved due to findings of typical MPAN symptoms in patients homozygous for this variant [1], our findings suggest that brain iron accumulation is less common, at least in early stages of the disease, in patients homozygous for c.187 G > C, p.(Ala63Pro).

Although there is currently no established clinical difference between patients homozygous for c.187 G > C, p.(Ala63Pro) compared to patients homozygous for more common variants, e.g., c.204_214del, p.(Gly69Argfs*10), our results, as well as previously described cases in the literature [14, 24], indicate a potential phenotypical difference that might be attributed to the causative genotype.

A potential pathophysiological mechanism for this phenotypical difference might be explained by whether the *C19orf12* variant causes membrane mis-localization of the protein or not.

The relatively small size of the *C19orf12* gene likely allows alleles with pathogenic variants to evade nonsense-mediated decay (NMD), a cellular quality control mechanism that normally eliminates mRNA transcripts containing premature stop codons. As a result, frame-shift and nonsense variants in *C19orf12* are probably translated into truncated proteins rather than being destroyed at the mRNA level, allowing these abnormal proteins to persist and cause disease.

Disease-causing variants affecting the transmembrane domain have been shown to lead to mis-localization of the protein from the membrane to cytosol. While this mis-localization has not been proved for c.204_214del, p.(Gly69Argfs*10) nor c.187 G > C, p.(Ala63Pro), it has been observed for c.172 G > A, p.(Gly58Ser), which is also predicted to be located in the transmembrane domain. This mis-localization of the protein in cells harboring this variant is assumed to be due to a glycine being replaced by serine, which changes the polarity of the transmembrane domain, making the protein unable to be correctly placed in the membrane [25].

Known pathogenic missense variants associated with MPAN in the transmembrane domain often involve amino acid changes of a glycine to more charged amino acids (e.g. p.(Gly53Arg), p.(Gly65Glu) and p.(Gly69Arg)). Furthermore, the glycines in these locations show high evolutionary conservation across multiple species, indicating a potential important molecular function [2].

The c.204_214del, p.(Gly69Argfs*10) variant involves a glycine and predicts a premature stop codon, leading to a truncated protein, while the c.187 G > C, p.(Ala63Pro) missense variant does not result in a prematurely terminated protein, nor does the predicted protein effect (Ala63Pro) involve a glycine, making protein mis-localization less likely.

The result might be a mis-localization of the protein to the cytosol, when both alleles are affected by c.204_214del, p.(Gly69Argfs*10), while the protein remains in the membrane, however, with some dysfunction, when both alleles are affected by c.187 G > C, p.(Ala63Pro). This could explain the seemingly milder phenotype of the c.187 G > C, p.(Ala63Pro) variant.

If this hypothesis is true, it would be expected, that patients homozygous for c.172 G > A, p.(Gly58Ser), which has been shown to result in protein mis-localization, would have a phenotype comparable to patients homozygous for c.204_214del, (p.Gly69Argfs*10).

To the best of our knowledge, only three patients homozygous for c.172 G > A, p.(Gly58Ser) have previously been described. The age at onset between these patients ranged between seven to 13 years of age. Symptoms included gait difficulties and optic atrophy. The patient with the youngest age at onset showed a mild cognitive deficit and brain iron accumulation at age 13, while the other two had normal cognition [26, 27]. Despite phenotypical variability among the three patients, their disease progression still aligns quite well with other described cases of homozygous c.204_214del, p.(Gly69Argfs*10) patients, in terms of age at onset, cognitive impairment and iron accumulation, suggesting that protein mis-localization might occur in both variants.

This potential protein mis-localization could be a possible molecular explanation for the seemingly milder phenotype for c.187 G > C, p.(Ala63Pro) compared to c.204_214del, p.(Gly69Argfs*10).

### A C19orf12-spectrum-disease

Panthothenate kinase associated neurodegeneration (PKAN), one of the most common types of NBIA, was previously named Hallervorden-Spatz syndrome. The condition was renamed to PKAN, because of discoveries about the disease’s underlying biochemistry and implicated gene [28]. Similarly, Behr syndrome, a disorder defined in 1909, seems to be almost clinically indistinguishable from MPAN [29].

These examples display how new discoveries continuously redefine diseases at a higher resolution, increasing accuracy and specificity, often leading to subclassifications. This is also the case with pathogenic *C19orf12* variants, which has led to the definition of SPG43, which is typically caused by c.187 G > C, (p.Ala63Pro). However, as more patients harboring this variant and showing classic MPAN symptoms were identified, the existence of the SPG43 phenotype was not supported, but instead seemed to diminish.

Despite this discovery, many of the described patients with homozygosity for c.187 G > C, (p.Ala63Pro), both in the literature and in this study, do seem to present with a somewhat milder phenotype, where cognitive decline is not as common compared to other variants.

While such minor differences in clinical findings do not seem to justify a completely new definition of a disease, it does show the clinical width of the phenotypical spectrum in *C19orf12*-related diseases. Consequently, it might be more informative and operational to classify MPAN phenotypes as a continuum ranging from mild to severe, rapidly progressing to slowly progressing, with or without brain iron accumulation, i.e., *C19orf12*-spectrum-disease, rather than belonging to the group of NBIAs.

### Limitations

*Prevalence estimation:* The prevalence of *C19orf12*-related disease in Denmark may be underestimated due to undiagnosed or misdiagnosed cases. Diagnostic delays are significant (five years for patient 1 and two years for patient 3) suggesting other patients may currently be navigating this prolonged diagnostic process. Misdiagnosis is a real concern given the rarity and broad phenotypical spectrum of the disease. There are reports of patients initially being misdiagnosed with frontotemporal dementia [10], Parkinson’s disease [2] and juvenile ALS [30], who were later identified as having pathogenic *C19orf12* variants.

*Patient comparability:* The comparability of the patients is challenged by significant age differences. Patients 1 and 2 are considerably younger than patients 3 and 4, from which it is reasonable to assume that patients 3 and 4 are at a later stage of disease compared to patients 1 and 2. This discrepancy complicates direct comparisons and conclusions about disease progression. However, when comparing patient 1 and 3, the duration of disease is quite similar at 9 and 8 years, respectively, while the clinical and imaging findings are still vastly different.

*Genotype-phenotype correlations and pathophysiology:* While our results suggest phenotypical differences between variants in *C19orf12*, it is difficult to draw definitive conclusions due to the few identified patients with homozygosity for c.187 G > C, p.(Ala63Pro). In addition to identifying more patients, functional studies of cells harboring different variants are required to further elucidate potential genotype-phenotype correlations and molecular functions. The proposed mechanism of protein mis-localization has not been proven for c.204_214del, p.(Gly69Argfs*10) nor c.187 G > C, p.(Ala63Pro), making our pathophysiological explanations hypothetical until further functional studies are conducted.

### Perspectives

The absence of iron accumulation, as seen in patients 1 and in the early disease stages of patient 2, raises questions regarding disease progression and development of brain iron accumulation. It is possible that patients 1 and 2 will experience cognitive decline and that patient 1 will develop brain iron accumulation at a later stage of disease; however, it is uncertain whether or when this occurs. Iron accumulation has been shown to be absent in early stages and present at later stages of disease which may be genotype dependent [30–33]. Despite iron accumulation being almost pathognomonic for MPAN, it has been suggested that the accumulation is an epiphenomenon that occurs as a sign of tissue degeneration and not a cause of disease [34, 35]. Iron accumulation occurs in many other neurodegenerative disorders and as an idiopathic age phenomenon, making it an unspecific finding [36]. Additionally, most of the genes involved in NBIA disorders, including *C19orf12*, have not been shown to be directly involved in iron homeostasis [37].

The unclear progression of brain iron accumulation in MPAN can be attributed to several factors. Firstly, diagnostic delays, which averages five years [38], result in MRIs being done well after symptom onset. Secondly, patients typically only receive one diagnostic MRI scan and are subsequently followed clinically rather than radiologically. Thirdly, in many studies, particularly in the initial descriptions of MPAN, patients were recruited based on pre-existing brain iron accumulation on MRI, creating selection bias [2, 12, 39, 40]. This approach, while effective for initially identifying cases of this ultra-rare disease, potentially overlooks an entire subset of patients with pathogenic *C19orf12* variants who lack iron accumulation. Patients homozygous for c.187 G > C, p.(Ala63Pro) (like patients 1 and 2) appear less likely to develop brain iron deposits and such patients might have been systematically excluded from previous research cohorts. This exclusion might distort our understanding of the full MPAN phenotypic spectrum and the prevalence of specific variants.

With growing awareness, ongoing discovery of novel MPAN cases and variants, and increasingly sophisticated diagnostic methods, more patients will likely be identified in the future. Denmark’s implementation of WGS-based diagnostics in clinical practice as of 2022 represents a paradigm shift from phenotype-driven to genotype-driven patient identification. This more agnostic approach has made comprehensive genetic evaluation more accessible for patients with suspected neurogenetic disorders. By focusing primarily on pathogenic variants rather than clinical or radiological presentations, this methodology will likely accelerate diagnosis, reduce the lengthy diagnostic delays typical for ultra-rare diseases, and diminish the general tendency to underdiagnose neurogenetic patients with atypical presentations. This WGS-based diagnostic approach may thereby expand our understanding of the clinical and radiological findings associated with pathogenic variants in *C19orf12*.

## Electronic supplementary material

Below is the link to the electronic supplementary material.

Supplementary material 1

Supplementary material 2

Supplementary material 3

Supplementary material 4

Supplementary material 5

## Data Availability

The sequencing data was generated as part of clinical testing, so the underlying raw data is consented for deposition to a public database.

## Abbreviations

MPAN: Mitochondrial membrane protein-associated neurodegeneration
NBIAs: Neurodegeneration with brain iron accumulation (disorders)
NBIA 4: Neurodegeneration with brain iron accumulation type 4 (another term for MPAN)
SPG-43: Spastic paraplegia type 43
HSP: Hereditary spastic paraplegia(s)
AR: Autosomal recessive
AD: Autosomal dominant
NGC: Danish National Genome Center
WGS: Whole-genome sequencing
WES: Whole exome sequencing
GENets: Genetic Expert Networks
MRI: Magnetic resonance imaging
PET: Positron emission tomography
PET-CT: Combined positron emission tomography and computed tomography
ADHD: Attention deficit hyperactivity disorder
FDG: ^18^F-fluorodeoxyglucose
PTSD: Post-traumatic stress disorder
CSF: Cerebrospinal fluid
EMG: Electromyography
ENG: Electroneurography
MND: Motor neuron disease
PE2I: ^18^F-FE-PE2I (a dopamine transporter ligand used in PET imaging)
GP: Globus pallidus
PKAN: Pantothenate kinase-associated neurodegeneration
NMD: Nonsense-mediated decay
ND: Not done
ALS: Amyotrophic lateral sclerosis
